# BLACK-WHITE RACIAL DISPARITIES IN MORTALITY: FINDINGS FROM THE SAINT LOUIS PERSONALITY AND AGING NETWORK (SPAN)

**DOI:** 10.1093/geroni/igae098.3811

**Published:** 2024-12-31

**Authors:** Isaiah Spears, Megan Wolk, Aaron Gorelik, Jayne Siudzinski, Sara Norton, Michael Esposito, Thomas Oltmanns, Ryan Bogdan

**Affiliations:** Washington University in St. Louis, St. Louis, Missouri, United States; Washington University in St. Louis, St. Louis, Missouri, United States; Washington University in St. Louis, St. Louis, Missouri, United States; Washington University in St. Louis, St. Louis, Missouri, United States; Washington University in St. Louis, St. Louis, Missouri, United States; University of Minnesota, Minneapolis, Minnesota, United States; Washington University in St. Louis, St. Louis, Missouri, United States; Washington University in St. Louis, St. Louis, Missouri, United States

## Abstract

Black-White racial health disparities in America are striking. Black Americans have worse physical health and lower life expectancy than White Americans. However, the potential factors contributing to increased mortality in later life among Black Americans are rarely investigated. Here, we examined Black-White racial mortality disparities in the longitudinal St. Louis Personality and Aging Network (SPAN) Study of older adults (n=1,630; aged 55-65 at baseline; 67.4% White; 32.6% Black), with mortality data collected from the National Death Index. Black SPAN participants had greater mortality over the course of the study than White participants(χ² = 47.667, p = <.0001). Specifically, 26.9% of Black participants (n = 142) passed away relative to 12.7% of White participants (n = 139). Indices of socioeconomic status (i.e., annual household income; educational attainment) partially accounted for this difference. Additional planned analyses will examine whether environmental (e.g., discrimination, stress exposure), biological (e.g., inflammation), and psychological/behavioral (e.g., health behaviors, personality) factors may also contribute to these Black-White mortality disparities. These findings replicate prior studies documenting racial health disparities, the potential contributions of socioeconomic status, and position the SPAN study to evaluate plausible environmental, behavioral, and biological mechanisms. Ultimately, better understanding the environmental, psychological/behavioral, and biological mechanisms that may contribute to Black-White racial health disparities may help facilitate interventions and policy to improve racial health equity.

